# An analysis of the influencing factors of false negative autoantibodies in patients with non-small cell lung cancer

**DOI:** 10.3389/fonc.2024.1358387

**Published:** 2024-05-10

**Authors:** Ailin Wang, Ying Hao, Yunlong Huo, Xiaoman Xu, Yi Zhang

**Affiliations:** ^1^ Department of Gerontology and Geriatrics, Sheng Jing Hospital of China Medical University, Shenyang, China; ^2^ Department of Pathology, Sheng Jing Hospital of China Medical University, Shenyang, China; ^3^ Department of Pulmonary and Critical Care Medicine, Sheng Jing Hospital of China Medical University, Shenyang, China

**Keywords:** autoantibody, non-small cell lung cancer, false negative, influencing factors, diagnosis

## Abstract

**Objectives:**

To analyze the clinical significance of seven autoantibodies (P53, PGP9.5, SOX2, GAGE7, GBU4-5, MAGE, and CAGE) in patients with non-small cell lung cancer (NSCLC) and the factors that influence false-negative results.

**Methods:**

Seven autoantibodies were measured in the serum of 502 patients with non-small cell lung cancer (NSCLC) using ELISA, and their correlations with age, sex, smoking history, pathological type, clinical stage, and PD-L1 gene expression were analyzed. The clinicopathological data of the false-negative and positive groups for the seven autoantibodies were compared to determine the influencing factors.

**Results:**

P53 antibody expression level was correlated with lobulation sign, PGP9.5 antibody expression level with sex and vascular convergence; SOX2 antibody expression level with pathological type, clinical stage, and enlarged lymph nodes; and MAGE antibody expression level with the pathological type (P<0.05). False-negative autoantibodies are prone to occur in lung cancer patients with ground-glass nodules, no enlarged lymph nodes, no vascular convergence, and PD-L1 gene expression <1% (P <0.05).

**Conclusion:**

Detection of seven autoantibodies was clinically significant in patients with NSCLC. However, poor sensitivity should be considered in clinical diagnoses to prevent missed diagnoses.

## 1 Introduction

Lung cancer, which manifests as malignant tumors in the lungs, has the highest incidence and fatality rate of all cancers in China, and is the most common cause of cancer-related deaths worldwide. Owing to its nonspecific clinical symptoms, early detection is challenging. Most patients with lung cancer are already at an advanced stage when diagnosed, and thus miss the best opportunity for treatment (1, 2). The 5-year survival rates differed significantly among patients with various NSCLC stages. Stage IA patients have a 5-year survival rate of 92% compared with only 6% for stage IV patients (3). Therefore, it is crucial to improve the early detection and identification of lung cancer. Early lung cancer screening is the most widely performed by low-dose computer tomography (LDCT). Studies have indicated that LDCT screening for lung cancer mortality in the high-risk group can be lowered by 20% (4–6), however, this method has a high false positive rate (>90% of nodules are found to be benign), which may result in additional unnecessary biopsies and other invasive surgeries, adding to the psychological stress of patients (7–9). It has been shown that LCDT can be combined with blood biomarkers to improve diagnostic precision and distinguish between benign and malignant nodules. The only blood biomarker that has been tested for prospective lung cancer screening is the autoantibody (AAB), which is 5 years sooner than computer tomography (CT) screening can detect lung cancer in the asymptomatic stage of the illness (10–13). The China Food and Drug Administration (CFDA) has recommended seven autoantibodies suitable for Chinese people (including p53, PGP9.5, SOX2, GAGE7, GBU4-5, MAGE, and CAGE), which have been confirmed by several studies (14–16) to have a positive clinical application value, however, their diagnostic sensitivity is low. The objective of this study was to investigate the clinical importance of autoantibodies in lung cancer, as well as the causes of false-negative results.

## 2 Materials and methods

### 2.1 Study population

A total of 502 newly diagnosed patients with lung cancer were recruited from the Shengjing Hospital of China Medical University between March 2021 and December 2022, including 268 males and 234 females, with 180 patients in the positive group and 322 patients in the false negative group. All patients were diagnosed by biopsy obtained after fiber bronchoscopy, percutaneous puncture, or surgery. None of the patients received antitumor therapy or chemoradiotherapy before the diagnosis of cancer, had complete medical history data, and had no other tumor history. This study was approved by the Ethics Committee of the Shengjing Hospital of China Medical University (Ethics number: 2022PS922K).

### 2.2 Autoantibody assay and cutoff

Serum from 3 to 5 ml fasting blood was separated by centrifugation at 3000 rpm for 10 min within 4 h of sample collection. Samples that could not be processed immediately were stored at −80 °C for future testing. An enzyme-linked immunosorbent assay (ELISA) was used according to the 7-AABS assay kit (Hangzhou Cancer Probe Biotech Company). The seven autoantibodies’ positive reference values were as follows: p53 ≥ 13.1 U/mL, PGP9.5 ≥ 11.1 U/mL, SOX2 ≥ 10.3 U/mL, GAGE7 ≥ 14.4 U/mL, GBU4-5 ≥ 7.0 U/mL, MAGE A1 ≥ 11.9 U/mL, and CAGE ≥ 7.2 U/mL. The result was considered positive if one of the seven autoantibodies tested positive. If all seven autoantibodies were negative, the samples were judged as negative.

### 2.3 Study method

**Figure 1:**
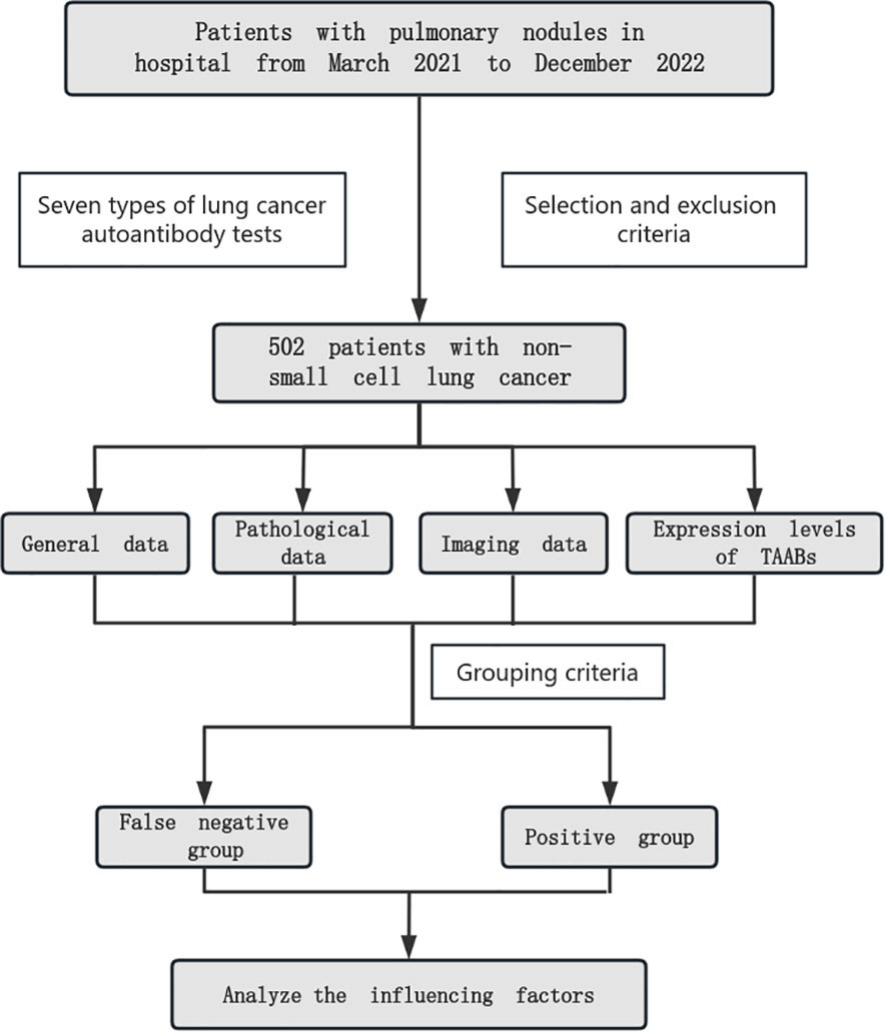
Specific flowchart of the research.

We collected general data, pathological data, imaging data and the expression levels of 7 auto antibodies from NSCLC patients who meet the inclusion criteria, analyzed the correlation between autoantibodies and general data, pathological data, imaging data and grouped them according to the cutoff of autoantibodies. Compared the clinical data of autoantibody false negative group and positive group, and further analyzed and identified the influencing factors of false negative (**Figure 1**).

### 2.4 Statistical analysis

SPSS26.0 software was used for statistical analysis. Since the level of sera of the seven autoantibodies (AABs) was not normally distributed, as determined by the Shapiro-Wilk’s test, the data were presented as medians (quartiles) (M(P25, P75]). The Mann–Whitney U test was used to compare the differences in antibody levels between two groups, and the Kruskal–Wallis H test was used to compare the differences in antibody levels among multiple groups. Counting data were expressed as n (%), the Chi-square test was used for comparison between groups, and binary logistic regression analysis was used to screen independent influencing factors of false negative autoantibody. A two-tailed P value lower than 0.05 was considered statistically significant.

## 3 Results

### 3.1 Relationship between lung cancer autoantibodies and the clinical data of patients with NSCLC

A detailed analysis of 502 lung cancer patients was conducted, and our results showed that age, smoking history, and PD-L1 gene expression had no impact on the expression level of 7-AABs detection. However, the expression level of the PGP9.5 antibody was noticeably higher in females than in males (P = 0.001). Squamous cell carcinoma patients had substantially higher SOX2 and MAGE antibody expression than adenocarcinoma patients (P = 0.002 and P = 0.03, respectively). In addition, Stage III lung cancer had considerably higher SOX2 antibody expression than those with Stage I lung cancer (P = 0.028) (**Table 1**).

**Table 1:** Relationship between lung cancer autoantibodies and clinical data of patients with NSCLC.

Group	n	P53	PGP9.5	SOX2	GAGE7	GBU4-5	MAGE	CAGE
		(U/ml)	(U/ml)	(U/ml)	(U/ml)	(U/ml)	(U/ml)	(U/ml)
Age (years)								
≤ 60	222	0.4(0.1~1.4)	0.1(0~0.2)	0.4(0.1~2.3)	0.65(0.2~2.75)	0.5(0.1~2.025)	0.1(0.1~0.2)	0.1(0~0.2)
> 60	280	0.4(0.2~1.2)	0.1(0~0.5)	0.4(0.1~2.425)	0.7(0.2~2.1)	0.4(0.1~1.975)	0.1(0~0.2)	0.1(0~0.2)
z/P		-0.313/0.754	-1.864/0.062	-0.457/0.648	-0.195/0.846	-0.598/0.55	-1.887/0.059	-0.641/0.521
Gender								
male	268	0.4(0.1~1.075)	0.1(0~0.2)	0.4(0.1~2.45)	0.6(0.2~2.1)	0.5(0.1~2.425)	0.1(0~0.2)	0.1(0~0.2)
female	234	0.45(0.2~1.5)	0.1(0~0.7)	0.4(0.1~2.225)	0.8(0.3~2.5)	0.4(0.1~1.9)	0.1(0~0.2)	0.1(0~0.2)
z/P		-1.493/0.135	-3.23/0.001^*^	-0.3/0.764	-1.319/0.187	-1.745/0.081	-1.004/0.315	-1.019/0.308
Smoking history								
never	292	0.4(0.1~1.2)	0.1(0~0.275)	0.4(0.1~1.975)	0.6(0.2~2.1)	0.5(0.1~1.975)	0.1(0~0.2)	0.1(0~0.2)
ever/current	210	0.4(0.2~1.4)	0.1(0~0.7)	0.5(0.1~3.35)	0.8(0.2~2.725)	0.3(0.1~2.025)	0.1(0~0.2)	0.1(0~0.1)
z/P		-1.009/0.313	-1.903/0.057	-1.461/0.144	-1.215/0.224	-1.408/0.159	-0.401/0.689	-0.934/0.35
Histology								
adenocarcinoma	431	0.4(0.2~1.3)	0.1(0~0.4)	0.4(0.1~2)	0.7(0.2~2.6)	0.5(0.1~1.9)	0.1(0~0.2)	0.1(0~0.2)
squamous cell carcinoma	71	0.4(0.2~2)	0.1(0~0.5)	1.1(0.1~6.7)	0.7(0.3~1.9)	0.4(0.1~2.9)	0.1(0.1~0.4)	0.1(0~0.1)
z/P		-0.552/0.581	-0.832/0.405	-3.071/0.002^*^	-0.317/0.751	-0.163/0.87	-2.936/0.03^*^	-0.679/0.497
Clinical stage								
I	322	0.4(0.175~1.1)	0.1(0~0.3)	0.3(0.1~2)*	0.6(0.2~1.9)	0.5(0.1~2.1)	0.1(0~0.2)	0.1(0~0.2)
II	75	0.4(0.2~1.3)	0.1(0~0.6)	0.4(0.1~2)	0.8(0.3~1.9)	0.3(0~1.2)	0.1(0.1~0.4)	0.1(0~0.1)
III	79	0.4(0.1~1.6)	0.1(0~0.7)	1(0.1~4.1)	0.8(0.2~5.1)	0.4(0.1~2.4)	0.1(0~0.2)	0.1(0~0.3)
IV	26	0.75(0.275~1.425)	0.1(0~0.325)	0.6(0.1~2.05)	1.1(0.3~5.8)	0.75(0.1~3.75)	0.1(0~0.225)	0.1(0~0.475)
H/P		3.418/0.332	1.244/0.743	8.236/0.041^*^	5.99/0.112	5.376/0.146	5.5/0.139	1.973/0.578
PD-L1gene								
<1%	287	0.5(0.2~1.4)	0.1(0~0.4)	0.4(0.1~1.8)	0.6(0.1~2.1)	0.5(0.1~2)	0.1(0~0.2)	0.1(0~0.2)
1-49%	183	0.3(0.1~1)	0.1(0~0.4)	0.5(0.1~3.2)	0.7(0.3~3.2)	0.4(0.1~2.5)	0.1(0~0.2)	0.1(0~0.2)
≥50%	32	0.3(0.125~1.375)	0.1(0~0.2)	0.55(0.1~8.8)	0.95(0.3~2.1)	0.35(0.1~1.5)	0.1(0.1~0.475)	0.1(0~0.175)
H/P		3.974/0.137	1.052/0.591	2.598/0.273	5.426/0.066	2.552/0.279	4.751/0.093	0.361/0.835

* Clinical stage I vs. Clinical stage III, P = 0.028.

### 3.2 Relationship between lung cancer AAbs and CT malignant signs of patients with NSCLC

Patients with enlarged lymph nodes had significantly higher SOX2 antibody production levels than patients without enlarged lymph nodes (P = 0.01). We analyzed 397 lung cancer patients with nodules smaller than 3 cm on CT. PGP9.5 and SOX2 antibody expression levels were significantly increased in patients with vascular convergence signs compared to patients without vascular convergence signs (P = 0.011, P = 0.012). Compared to patients without lobulation, patients with lobulation had substantially higher levels of p53 antibody expression (P = 0.03). There was no significant difference in the expression of the seven AAbs among different tumor sizes, nodule types, vacuole signs, spiculation signs, and pleural indentations (P>0.05) (**Table 2**).

**Table 2:** Relationship between autoantibodies and CT malignant signs of patients with NSCLC.

Group	n	p53	PGP9.5	SOX2	GAGE7	GBU4-5	MAGE	CAGE
		(U/ml)	(U/ml)	(U/ml)	(U/ml)	(U/ml)	(U/ml)	(U/ml)
Tumor size								
≤ 8mm	16	0.95(0.2~3.2)	0.1(0~0.45)	1.3(0.1~4.7)	0.45(0.125~0.975)	0.7(0.1~2.9)	0.15(0.025~1.125)	0.1(0.025~0.75)
9-20mm	238	0.4(0.2~1.2)	0.1(0~0.425)	0.5(0.1~2.55)	0.6(0.2~1.8)	0.5(0.1~1.8)	0.1(0~0.2)	0.1(0~0.2)
> 20mm	248	0.4(0.1~1.3)	0.1(0~0.3)	0.4(0.1~1.975)	0.8(0.2~3.275)	0.4(0.1~2.35)	0.1(0~0.2)	0(0~0.2)
H/P		2.047/0.359	0.293/0.864	1.277/0.528	2.023/0.364	0.191/0.909	1.857/0.395	2.122/0.346
Types								
Pure GGN	115	0.4(0.1~1.3)	0.1(0~0.4)	0.3(0.1~1.3)	0.5(0.1~1.6)	0.6(0.1~1.9)	0.1(0~0.2)	0.1(0~0.2)
Solid GGN	225	0.4(0.2~1.3)	0.1(0~0.4)	0.5(0.1~2.25)	0.7(0.2~2.6)	0.4(0.1~1.9)	0.1(0~0.2)	0.1(0~0.2)
Mixed Nodules	162	0.4(0.2~1.125)	0.1(0~0.3)	0.5(0.1~3.475)	0.65(0.3~2.65)	0.4(0.1~2.925)	0.1(0~0.2)	0(0~0.2)
H/P		0.434/0.805	0.028/0.986	3.459/0.177	2.786/0.248	1.367/0.505	2.75/0.253	1.377/0.502
Enlarged Lymph nodes								
none	332	0.4(0.2~1.175)	0.1(0~0.3)	0.35(0.1~1.9)	0.7(0.2~2.1)	0.5(0.1~1.9)	0.1(0~0.2)	0.1(0~0.2)
yes	170	0.4(0.1~1.7)	0.1(0~0.6)	0.6(0.1~4.075)	0.6(0.2~2.3)	0.4(0.1~2.525)	0.1(0~0.2)	0.1(0~0.1)
z/P		-0.84/0.401	-1.457/0.145	-2.561/0.01^*^	-0.488/0.626	-0.234/0.815	-0.337/0.736	-1.019/0.308
Vascular convergence								
none	240	0.4(0.2~1.2)	0.1(0~0.375)	0.3(0.1~1.7)	0.55(0.2~1.7)	0.4(0.1~1.975)	0.1(0~0.2)	0.1(0~0.2)
yes	157	0.4(0.2~1.3)	0.1(0~0.6)	0.7(0.1~3.6)	0.8(0.2~3.75)	0.5(0.1~1.95)	0.1(0.1~0.2)	0.1(0~0.2)
z/P		-0.193/0.847	-2.535/0.011^*^	-2.523/0.012^*^	-1.894/0.058	-0.383/0.702	-0.614/0.539	-1.495/0.135
Vacuole sign								
none	355	0.4(0.2~1.2)	0.1(0~0.4)	0.4(0.1~2.7)	0.6(0.2~2.1)	0.4(0.1~1.9)	0.1(0~0.2)	0.1(0~0.2)
yes	42	0.45(0.2~1.35)	0.1(0~0.6)	0.15(0.1~1.175)	0.8(0.2~1.925)	0.45(0.1~3.275)	0.1(0.1~0.2)	0.1(0~0.2)
z/P		-0.138/0.89	-0.536/0.592	-1.412/0.158	-0.202/0.84	-0.467/0.64	-1.369/0.171	-0.82/0.412
Lobulation sign								
none	328	0.4(0.1~1.175)	0.1(0~0.475)	0.4(0.1~2.975)	0.6(0.2~2.1)	0.5(0.1~1.9)	0.1(0~0.2)	0.1(0~0.2)
yes	69	0.5(0.2~1.55)	0.1(0~0.45)	0.4(0.1~1.1)	0.6(0.2~1.45)	0.3(0.1~2.15)	0.1(0.1~0.25)	0.1(0~0.2)
z/P		-2.167/0.03^*^	-0.464/0.643	-0.547/0.584	-0.169/0.865	-0.761/0.447	-1.17/0.242	-0.131/0.896
Spiculation sign								
none	312	0.4(0.1~1.175)	0.1(0~0.4)	0.4(0.1~2.275)	0.6(0.2~1.9)	0.45(0.1~1.9)	0.1(0~0.2)	0.1(0~0.2)
yes	85	0.4(0.2~1.5)	0.1(0~0.65)	0.5(0.1~3)	0.6(0.2~3.35)	0.4(0.1~2.7)	0.1(0.05~0.2)	0.1(0~0.2)
z/P		-0.999/0.318	-1.089/0.276	-0.676/0.499	-0.089/0.929	-0.831/0.406	-0.267/0.789	-1.339/0.181
Pleural depression								
none	280	0.4(0.1~1.1)	0.1(0~0.4)	0.4(0.1~2.275)	0.6(0.2~1.975)	0.45(0.1~1.9)	0.1(0~0.2)	0.1(0~0.2)
yes	117	0.4(0.2~1.55)	0.1(0~0.55)	0.5(0.1~3.05)	0.6(0.2~2.1)	0.4(0.1~2.35)	0.1(0~0.2)	0.1(0~0.2)
z/P		-0.815/0.415	-0.732/0.464	-0.31/0.757	-0.647/0.518	-0.95/0.342	-0.013/0.99	-0.534/0.594

* GGN, ground glass nodule.

### 3.3 Univariate analysis of false negative autoantibodies in lung cancer

To investigate the influencing factors of false-negative autoantibodies, we performed univariate analysis to compare clinicopathological data of the false-negative and positive groups for the seven AAbs. False-negative autoantibodies in lung cancer may be influenced by smoking history, pathological type, PD-L1 expression, nodular type, and enlarged lymph nodes (P < 0.05). Age, sex, clinical stage, and tumor size did not differ significantly between the two groups (P > 0.05) (**Table 3**).

**Table 3:** Comparison of clinical data between false negative and positive patient groups.

	False negative group(n = 321)	Positive group(n = 181)	X^2^	P
≤ 60 years [n(%)]	140 (43.6)	82 (45.3)	0.134	0.714
Female [n(%)]	173 (53.9)	95 (52.5)	0.092	0.761
Smoking history [n(%)]	120 (37.4)	90 (49.7)	7.244	0.007^*^
Adenocarcinoma [n(%)]	284 (88.5)	147 (81.2)	5.201	0.025^*^
Stage I+I [n(%)]	262 (81.6)	135 (74.6)	3.462	0.063
PD-L1gene [n(%)]			7.443	0.024^*^
< 1%	197 (61.4)	90 (49.7)		
1-49%	103 (32.1)	80 (44.2)		
≥ 50%	21 (6.5)	11 (6.1)		
Tumor size [n(%)]			5.775	0.056
≤8 mm	10 (3.1)	6 (3.3)		
9-20 mm	165 (51.4)	73 (40.3)		
>20 mm	146 (45.5)	102 (56.4)		
Types [n(%)]			8.481	0.014^*^
Pure GGN	86 (26.8)	29 (16.0)		
Solid GGN	141 (43.9)	84 (46.4)		
Mixed nodules	94 (29.3)	68 (37.6)		
Enlarged lymph nodes [n(%)]	89 (27.7)	81 (44.8)	14.98	< 0.001^*^

* GGN, ground glass nodule.

The vascular convergence and spiculation signs in the malignant signs shown by CT may be possible influencing factors of false-negative autoantibodies in lung cancer (P<0.05). There were no significant differences in vacuole sign, lobulation sign, or pleural indentation between the two groups (P > 0.05) (**Table 4**).

**Table 4:** Comparison of CT malignant signs between false negative and positive patients groups.

	False negative group(n=257)	Positive group(n=140)	X^2^	P
Vascular convergence [n(%)]	79 (30.7)	78 (55.7)	23.646	< 0.001^*^
Vacuole sign [n(%)]	28 (10.9)	14 (10.0)	0.077	0.782
Lobulation sign [n(%)]	44 (17.1)	25 (17.9)	0.034	0.853
Spiculation sign [n(%)]	42 (16.3)	43 (30.7)	11.125	0.001^*^
Pleural depression [n(%)]	70 (27.2)	47 (33.6)	1.749	0.186

*P<0.05.

### 3.4 Multivariate analysis of false negative autoantibodies in patients with lung cancer

Binary logistic regression analysis showed that PD-L1 gene expression < 1%, Pure GGN, absence of enlarged lymph nodes, and vascular convergence were all independent influencing factors of false-negative autoantibodies in lung cancer (P<0.05) (**Table 5**).

**Table 5:** Multivariate analysis of false negative autoantibodies in patients with lung cancer.

Influencing factors	b	Standard error	Wald X^2^	Odds ratio (95% CI)	p
Smoking history	0.234	0.213	1.211	1.264 (0.833~1.92)	0.271
squamous cell carcinoma	0.128	0.308	0.172	1.136(0.621~2.079)	0.679
PD-L1 gene					
1-49%	0.478	0.209	5.256	1.613 (1.072~2.428)	0.022^*^
≥ 50%	-0.181	0.427	0.179	0.834 (0.361~1.928)	0.672
Types					
Solid GGN	0.390	0.272	2.048	1.477 (0.866~2.519)	0.152
Mixed nodules	0.766	0.275	7.761	2.151 (1.255~3.687)	0.005^*^
Enlarged lymph nodes	0.704	0.210	11.252	2.022 (1.340~3.050)	0.001^*^
Vascular convergence	0.914	0.228	16.113	2.495 (1.597~3.9)	< 0.001^*^
Spiculation sign	0.499	0.265	3.533	1.647 (0.979~2.77)	0.06

*P<0.05.

## 4 Discussion

The establishment of a clinical pathway for early lung cancer screening is essential for improving the prognosis and early detection of lung cancer in patients. In the early stage of cancer, the human immune system can produce autoantibodies against tumor cells through humoral immunity, which provides a theoretical basis for the application of autoantibodies in the early screening of lung cancer (17, 18). Several studies have shown that the sensitivity of the combined detection of the seven autoantibodies is higher than that of individual antibody detection (14, 19–21). Chapman et al. (22) studied seven autoantibodies (p53, c-myc, HER-2, NYESO1, CAGE, MUC1, and GBU4-5) in Europeans with a sensitivity of 76% and a specificity of 92%. In China, Ren (16) et al. used seven autoantibodies (p53, PGP9.5, SOX2, GAGE7, GBU4-5, MAGE, and CAGE) with a sensitivity and specificity of 61% and 90%, respectively. According to Zang’s theory (23), the ideal combination of two autoantibodies, Annexin A1-Ab, and Alpha enolase-Ab, with CEA and CA125 could also greatly increase diagnostic effectiveness. However, finding the ideal autoantibody combination remains a current study focus for the early detection of lung cancer. This combination should have high sensitivity and specificity, and be closely linked to the clinicopathological characteristics of patients to play a better role in the early screening, diagnosis, and prognosis of lung cancer.

In this study, we only studied patients with non-small cell lung cancer and did not set a control group comprising healthy individuals or patients with benign lung conditions, which has certain limitations, and we will further refine the study and set up a control group in the future. The autoantibody positivity rate in patients with lung cancer was found to be 36.05%. The reasons for this may be as follows: the sample size was limited because only the findings of lung cancer patients with PD-L1 gene detection were included in this study, which had a small sample size. Furthermore, Many individuals had lung cancer in its early stages. 397 patients had stage I + II lung cancer, accounting for 79.08% of all patients included in this study. Finally, the critical value of autoantibodies in this study was determined according to the standards recommended by seven autoantibody detection kits from Hangzhou Kaipao Biotechnology Co., Ltd., which may not be completely applicable to the local population. More clinical studies should be conducted to determine the threshold of autoantibodies for local lung cancer patients.

Through analysis of the clinical data and pathological features of patients with lung cancer, it was found that lung cancer autoantibodies have the following characteristics: PGP9.5 antibody expression levels were noticeably higher in females than in males. According to Li et al. (24), the expression level of the p53 antibody was much higher in males than in females, but Mu (25) discovered that the sex of the patients did not influence the expression levels of the seven autoantibodies. The results of these clinical studies were different, which may be related to a different selection of cases, population differences, and diverse detection methods. The expression level of SOX2 antibody and MAGE antibody in squamous cell carcinoma was significantly higher than that in adenocarcinoma, and the expression level of SOX2 antibody in stage III lung cancer was significantly higher than that in stage I lung cancer. It is reported that SOX2 antibody is expressed in different parts of squamous cell carcinoma. According to a study by Brcic et al. on 146 NSCLC cases, squamous cell carcinoma (72%) expressed SOX2 much more than adenocarcinoma (8%), and the overexpression of SOX2 may be associated with a good prognosis for squamous cell carcinoma (26–28). Luo (29) et al. found that squamous cell carcinomas had a much higher rate of MAGE antibody positivity than adenocarcinomas. Among the 105 pairs of lung adenocarcinoma and pericancerous tissues, Sang (30)et al. discovered that 46.66% of lung adenocarcinoma specimens expressed the MAGE family, which was associated with a low 10-year survival rate and poor prognosis, suggesting that SOX2 and MAGE antibodies could be used as markers to classify various histological subtypes of lung cancer and assess their prognosis.

The statistically significant factors between the lung cancer autoantibody false-negative group and the positive group were analyzed by binary logistic regression analysis and the probability of false-negative autoantibodies in patients with PD-L1 gene expression < 1% was 1.613 times higher than PD-L1 gene expression of 1-49%. Patients with ground-glass nodules had a 2.151 times higher probability of developing false-negative autoantibody results than those with mixed nodules. The probability of false-negative autoantibodies in patients without lymph node enlargement was 2.022 times higher than that in patients with lymph node enlargement. The probability of false-negative autoantibodies in patients without a vascular convergence sign in the nodules was 2.495 times higher than that in patients with a vascular convergence sign. Some studies have shown that the proportion of solid components in nodules is highly correlated with tumor invasiveness, a key indicator of tumor malignancy (31). This might be related to the fact that the mixed nodules in this study were more likely to exhibit positive autoantibodies. Researchers in China have investigated the connection between lung cancer autoantibodies and imaging characteristics. While Meng et al. suggested that the positive expression of lung cancer autoantibodies was only related to pleural indentation, Jia et al. discovered a correlation between a short burr and the vacuole sign. Although the aforementioned findings vary, they demonstrate that the presence of autoantibodies correlates with the malignant symptoms of lung cancer, which is useful for differentiating benign from malignant pulmonary nodules. As there are not many clinical research reports in this area in China right now, we can look for multicenter collaboration to increase the sample size and further investigate the connection between lung cancer autoantibodies and CT symptoms. In recent years, immunotherapy represented by PD-1/PD-L1 inhibitors has made a breakthrough in the treatment of lung cancer, and several clinical studies have shown that patients with high levels of PD-L1 gene expression in tissues are better treated with anti-PD-1/PD-L1 inhibitors (32, 33). Several studies have also confirmed that positive autoantibodies are associated with greater efficacy and longer survival time following immunotherapy (34). However, whether autoantibodies may become predictive biomarkers of immunotherapy need to be proven by additional experiments.

Overall, the detection of the seven autoantibodies in patients with NSCLC has certain supplementary effects in the clinic, but its sensitivity is modest. More consideration should be given to how the aforementioned factors (ground glass nodules, no enlarged lymph nodes, and no vascular convergence sign) affect the outcomes of the examination when lung cancer autoantibodies are tested in patients with suspected but not fully diagnosed lung cancer. Further clinical techniques should be used to aid in diagnosis based on the real circumstances of the patients to lower the incidence of missed diagnoses, increase the detection rate, and carry out an early clinical intervention.

## Data availability statement

The raw data supporting the conclusions of this article will be made available by the authors, without undue reservation.

## Ethics statement

The studies involving humans were approved by Ethics Committee of the Shengjing Hospital of China Medical University. The studies were conducted in accordance with the local legislation and institutional requirements. The participants provided their written informed consent to participate in this study.

## Author contributions

AW: Writing – original draft, Investigation, Data curation. YHa: Writing – review & editing, Software, Methodology, Investigation. YHu: Writing – review & editing, Validation, Supervision, Resources. YZ: Writing – review & editing, Visualization, Supervision, Project administration. XX: Writing – review & editing, Validation, Supervision, Project administration, Conceptualization.
